# Leukocytoclastic Vasculitis as a Rare Manifestation of Staphylococcal Osteomyelitis

**DOI:** 10.7759/cureus.15685

**Published:** 2021-06-16

**Authors:** Sravani Lokineni, Amr Mohamed, Roopali Goyal Gandhi, Mary Barrett

**Affiliations:** 1 Internal Medicine, Rochester Regional Health, Rochester, USA; 2 Dermatopathology, Rochester Regional Health, Rochester, USA

**Keywords:** small-vessel vasculitis, osteomyelitis, infection, leukocytoclastic vasculitis, staphylococcus aureus

## Abstract

Leukocytoclastic vasculitis (LCV), also known as small-vessel cutaneous vasculitis, is rarely seen in the setting of staphylococcal infection without bacteremia. Here, we present a case of LCV in a 61-year-old male with chronic staphylococcal osteomyelitis without any evidence of bacteremia. The rash resolved with the treatment of osteomyelitis. As seen in this case, local infection should be considered in the differential diagnosis of LCV even when there is no evidence of bacteremia.

## Introduction

Leukocytoclastic vasculitis (LCV) is characterized by the inflammation of small cutaneous blood vessels in the setting of various triggering agents such as infection, drugs, and connective tissue diseases [1]. It frequently presents as palpable purpuric lesions and can lead to disabilities. Most available literature describes LCV to occur in the setting of bacteremia. However, no cases have been reported regarding LCV occurring with local infection or with osteomyelitis. This report highlights the importance of recognizing specific etiologic agents, especially infections, even without any evidence of bacteremia, as early treatment of infection can result in the resolution of LCV and reduce morbidity.

## Case presentation

**Figure 1:**
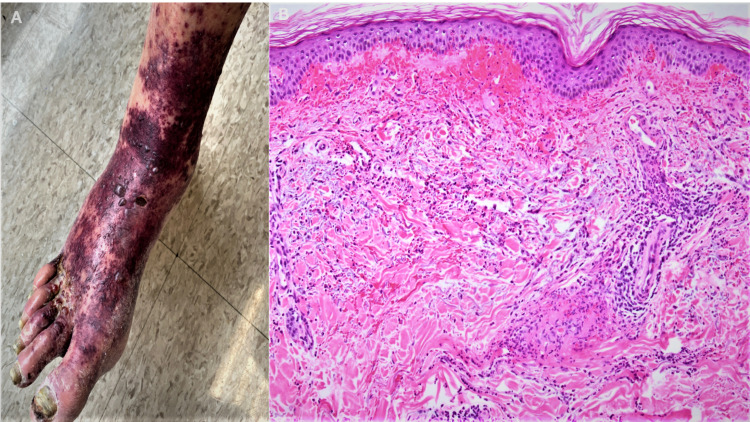
(A) Petechial skin eruptions on the right dorsum of the foot and mid-leg with hemorrhagic bullae and chronic stasis skin changes. (B) Skin with dermal hemorrhage, perivascular and interstitial neutrophils with leukocytoclasis, and focal fibrinoid necrosis of small blood vessels (H&E 100×). H&E: hematoxylin and eosin

A 61-year-old male with a history of peripheral vascular disease and uncontrolled diabetes mellitus presented to the hospital with a non-healing left foot ulcer without fever or chills. This was associated with a new onset of a generalized, purple, non-itchy, and non-painful skin rash of three-day duration. The rash initially started on his lower extremities and subsequently generalized. He denied any prior history of a similar rash and autoimmune connective tissue diseases. He also denied recent antibiotic usage. The patient’s vital signs were normal; however, the physical examination showed palpable, non-blanchable petechial skin changes with hemorrhagic bullae (Figure 1A) on his feet, legs, arms, back, and flanks, along with a non-healing ulcer under the left big toe.

As magnetic resonance imaging (MRI) of the feet could not be performed because of an implantable defibrillator, we performed a bone scan with evidence of left first metatarsal head osteomyelitis. The patient underwent debridement and bone resection of the left first metatarsal bone. Bone cultures grew methicillin-sensitive *Staphylococcus aureus* (MSSA) (1+), group B *Streptococcus* (rare), and *Corynebacterium* (2+). The patient was initially started on cefazolin and then shifted to a two-dose regimen of dalbavancin after three days. Although cefazolin was the best option to treat MSSA, as *Corynebacterium* was the most prevalent organism in the bone culture, the patient was started on dalbavancin to cover MSSA, group B *Streptococcus*, and *Corynebacterium*.

We also performed a complete vasculitis workup, including anti-neutrophil cytoplasmic antibody (ANCA), anti-nuclear antibody (ANA), and serum electrophoresis, which were all negative. Infectious workup for hepatitis B, hepatitis C, and HIV was negative. Blood cultures were negative as well. In addition, skin biopsy of the left forearm showed mild to moderately dense superficial perivascular and interstitial neutrophilic infiltrate with leukocytoclasis, focal fibrinoid necrosis, and numerous extravasated erythrocytes, which was consistent with LCV (Figure 1B). The rash significantly improved upon treatment of the infection with intravenous dalbavancin. Therefore, the rash was presumed to be due to MSSA infection.

## Discussion

LCV, also referred to as small-vessel vasculitis of the skin, is an inflammation of the small blood vessels [1], which presents as non-blanching palpable purpura and primarily affects the lower extremities. It occurs due to an immune complex deposition in the small blood vessels often in the setting of a triggering condition, such as certain drugs [1] or infections [2]. Typically, palpable purpura occurs seven to ten days after the onset of infection. According to the 2012 revised International Chapel Hill Consensus [3], small-vessel vasculitis is classified into the following categories: (1) ANCA-associated vasculitis; (2) complex immune vasculitis; (3) vasculitis associated with systemic diseases; and (4) vasculitis associated with an etiology (e.g., infections, drugs, and cancer).

In our case, the patient had chronic osteomyelitis due to non-healing ulcers on the left foot, which probably triggered an immune complex deposition in the small vessels. This condition has rarely been seen in the setting of MSSA infection without bacteremia. A previous study reported a case of LCV caused by MSSA bacteremia in a 34-year-old man with a purulent elbow wound, which resolved on treating the infection [4].

A similar case in the literature reported a 71-year-old woman with acute bacterial endocarditis and negative blood cultures presenting with LCV, which resolved with the treatment of the infection [5]. LCV has also been reported with *Klebsiella pneumoniae* bacteremia in a 62-year-old Taiwanese woman with disseminated pustulosis [6], as well as in association with group B meningococcal bacteremia [7], with resolution on the treatment of respective infections. A recent case-control study showed that LCV is a common dermatologic manifestation in patients with severe coronavirus disease 2019 infection [8].

If the etiological agent is unclear, a complete workup should be performed to look for any systemic causes [9]. Typically, the workup includes complement levels C3 and C4, ANCA, ANA, and serum electrophoresis. A skin biopsy is usually the most definitive diagnostic test for LCV, showing neutrophilic infiltrate in the superficial and mid dermal blood vessels along with granulocytic debris (leukocytoclasia), fibrinoid necrosis, and extravasation of red blood cells [9]. LCV usually resolves with the removal of triggering agents and on treating the infection. If the skin rash is severe and refractory, adjunctive therapies such as corticosteroids, colchicine, and dapsone can be helpful [9]. In the current case, the patient’s rash improved with intravenous dalbavancin treatment.

## Conclusions

This case emphasizes the importance of early definitive diagnosis of LCV by skin biopsy and finding the causative agent. If no specific cause has been defined, it is imperative to evaluate for secondary causes of vasculitis. This report stresses the importance of considering infection in the differential diagnosis when evaluating a case with LCV. Additionally, LCV can be a complication of a local infection even in the absence of bacteremia.
